# The possible relationship between the healthy eating index-2015 and the 10-year risk of cardiovascular diseases

**DOI:** 10.1186/s40795-023-00735-8

**Published:** 2023-06-28

**Authors:** Pegah Ahmadijoo, Mohammad Hassan Eftekhari, Seyed Jalil Masoumi, Maryam Ranjbar Zahedani, Farzaneh Mohammadi

**Affiliations:** 1 Department of Clinical Nutrition, School of Nutrition and Food Sciences, Shiraz University of Medical Sciences, Shiraz, Iran; 2 Nutrition Research Center, School of Nutrition and Food Sciences, Shiraz University of Medical Science, Shiraz, Iran; 3 Gastroenterohepatology Research Center, Shiraz University of Medical Sciences, Shiraz, Iran; 4 Department of Nutrition Sciences, School of Health, Larestan University of Medical Sciences, Larestan, Iran; 5 Student Research Committee, Shiraz University of Medical Sciences, Shiraz, Iran

**Keywords:** Body mass index, Cardiovascular disease, Framingham risk score, QRISK3, Healthy eating index

## Abstract

**Background:**

Cardiovascular diseases (CVDs) remain the leading cause of mortality worldwide. This underlies the need to evaluate different targets, such as diet quality. In this regard, we conducted the present study to find whether the healthy eating index-2015 (HEI-2015) score is associated with a 10-year risk of CVDs based on Framingham Risk Score (FRS) and QRISK3 in different body mass index (BMI) groups.

**Methods:**

This cross-sectional study was performed based on Shiraz University of Medical Sciences Employees Health Cohort Study (SUMS EHCS) data in April 2020. A total of 764 participants met the inclusion criteria. An expert performed demographic, anthropometric, and dietary evaluations. A semi-quantitative food frequency questionnaire (FFQ) was applied to assess the diet quality, and FRS and QRISK3 were used to evaluate the 10-year risk of CVDs.

**Results:**

Based on the results, many components of HEI-2015 indicated an increasing trend through quartiles (*p* < 0.001). However, the consumption of refined grains in higher quartiles showed a decreasing trend (*p* < 0.001). The consumption of added sugar and saturated fatty acids (SFAs) in higher quartiles revealed an increasing trend (*p* < 0.001). In addition, lower HEI-2015 scores and lower whole grain consumption were significantly associated with higher BMI (*p* < 0.05). Also, lower consumption of fruits showed a significant relationship with higher risk scores of Framingham and QRISK3 (*p* < 0.05). Higher added sugar and SFAs intake was significantly related to lower FRS (*p* < 0.05). A significant reverse association between HEI-2015 and QRISK3 and Framingham risk scores was seen (*p* < 0.05).

**Conclusion:**

Our findings support dietary recommendations to increase fruit and whole grains intake to prevent CVD and obesity. Moreover, a significant inverse association between HEI-2015 and QRISK3 and Framingham risk scores was observed. Since the results for added sugars and SFA intakes were controversial, further studies are needed.

## Introduction

Cardiovascular diseases (CVDs) are known as the leading cause of premature death globally [1]. Based on the world health organization (WHO) report, CVDs accounted for 17.3 million deaths worldwide in 2008, and it is predicted that this figure will increase to almost 23.6 million deaths by 2030 [2]. For decades, in Iran, the most widespread cause of death has been transferred from infectious diseases to CVDs and it accounts for 46% of all deaths [3].

Multivariate CVDs risk assessment tools are available to identify individuals at risk for effective preventive interventions including drug administration and lifestyle changes [4]. Obesity contributes to all causes of mortality, especially CVDs, by inducing destructive effects on cardiac structure and function [5, 6]. Moreover, the Framingham Risk Score (FRS), the most popular risk method, predicts the 10-year risk of cardiovascular events which has previously been confirmed to apply to the Asian population [7]. Also, QRISK3 is another reliable CVDs risk predictor which considers corticosteroid use, erectile dysfunction, anxiety, depression, and autoimmune disease, in addition to traditional risk factors [8]. However, there is a lack of evidence about the effect of dietary behaviors on total chronic disease risk.

Diet quality has been established as a major modifiable risk factor for preventing CVDs and all-cause mortality [9]. In this regard, the use of various diet-quality indices has become prevalent. The advantage of using such indices is evaluating overall diet rather than a single component, in the chronic diseases [10]. A healthy eating index (HEI) is suggested as an indicator of overall diet quality, which can predict people’s adherence to dietary guidelines [11]. HEI presents the complexity of dietary patterns and is negatively associated with the biomarkers of disease, while it is positively associated with the serum levels of vitamin A, folate, and vitamin C [11, 12]. The HEI-2015 was updated based on the most recent 2015–2020 Dietary Guidelines for Americans; according to previous findings, its higher scores were inversely related to mortality risk [13]. However, the possible association between HEI-2015 and the incidence of CVDs has not been evaluated properly.

Since CVDs have become one of the most widespread and important issues in the last few decades and only a few studies have evaluated the associations between diet quality and CVDs risk, the primary objective of our study was to find whether the HEI-2015 score is associated with a 10-year risk of CVDs based on FRS and QRISK3 scores.

## Material and methods

This cross-sectional study used data from the adult population who participated in Shiraz University of Medical Sciences Employees Health Cohort Study (SUMS EHCS) in April 2020. SUMS EHCS is an ongoing cohort which has started in 2017 and investigates health parameters in the employed population of SUMS who were visited at the cohort center in Motahari Clinic, Shiraz, Iran in accordance with the Helsinki Declarations of Ethics. In this study, the participants aged 40–74 years with a BMI of higher than 18.5 kg/m^2^, a total energy intake of 800–4200 kcal/day, no history of cardiovascular events, and no consumption of diabetes and lipid regulating medications were included.

All participants who joined the study signed a written informed consent and the ethics committee of Shiraz University of Medical Sciences approved our study (Code: IR.SUMS.REC.1399.482). Demographic, anthropometric, and dietary evaluations were performed by experts. Also, at the beginning of the study, participants were asked to record their current smoking status and current status of receiving the blood pressure medication.

### Anthropometric measurements

To measure the height and weight, we used a stadiometer to the nearest 0.1 cm and a standard scale (Seca, Germany) to the nearest 0.1 kg in participants without shoes and with light clothing. BMI was computed as weight (kg) divided by squared height (meter) [14]. Based on the WHO recommendation, the participants were grouped into the following categories: 18.5 ≤ BMI < 25 as normal weight, 25 ≤ BMI < 30 as overweight, and 30 ≤ BMI as obese [15].

### Blood pressure measurement

Blood pressure was performed by an experienced expert using a standard sphygmomanometer (Riester Precisa-N, Germany). Participants were rested in a sitting position for 10 min before blood pressure measurement. Then blood pressure was measured twice on the right and left arm at an interval of 10 min. The average of these multiple blood pressures was reported as the final number.

### Laboratory measurements

Blood sample was collected by a laboratory technician after 12 h of fasting. Plasma glucose levels, total cholesterol, and high-density lipoprotein cholesterol (HDL-C) were assayed by an enzymatic colorimetric methodology (Pars Azmoon., Tehran, IRAN).

### Framingham risk score (FRS) and Qrisk3

The FRS and QRISK3 equations for CVDs were used to predict the risk of an event. The FRS was estimated for each participant using the risk score based on age, gender, total cholesterol, HDL-C, systolic blood pressure, smoking, diabetes, and hypertension medication intake [16]. However, QRISK3 risk score includes ethnicity, age, gender, BMI, total cholesterol/high-density lipoprotein cholesterol ratio, systolic blood pressure, smoking, diabetes, hypertension medication intake, family history, chronic kidney diseases, migraine, rheumatoid arthritis, systemic lupus erythematosus, severe mental illness, erectile dysfunction, steroids, atypical antipsychotic medication, and atrial fibrillation [16]. To prevent errors, we only entered common variables into online calculators. The final scores were categorized into three levels: < 10% as low-risk, 10–20% as moderate risk, and > 20% as high risk of CVD [17].

### Dietary assessments

All data were collected by trained nutritionists in a face-to-face interview. A semi-quantitative food frequency questionnaire (FFQ) with 125 items was used to assess the participants' food intake on a daily, monthly, and annual basis. The validity and reliability of the questionnaire had been confirmed in previous studies [18]. Dietary intakes were analyzed and converted to grams by Nutritionist IV software (First Databank, San Bruno, CA, USA) modified for Iranian foods.

HEI-2015 was used for evaluating adherence to the healthy eating guidelines. HEI-2015 contains 13 components including total fruits, whole fruits, total vegetables, greens and beans, whole grains, refined grains, dairy products, total protein foods, seafood and plant proteins, fatty acids ratio, sodium, added sugars, and saturated fatty acids (SFA). Intake of total fruits, whole fruits, total vegetables, greens and beans, total protein foods, seafood, and plant proteins received 5 scores in the highest consumption, and the lowest intake scored 0. High consumption of whole grains, dairy, and fatty acids (polyunsaturated fatty acids (PUFAs) and monounsaturated fatty acids (MUFAs) to SFAs ratio) received 10, and the lowest intake of them was scored 0. Moreover, the intake of high amounts of refined grains, sodium, added sugars, and saturated fats had 0 points, while the lowest intake of them had 10 points. Finally, the scores were summated, and the HEI-2015 was scored from 0 to 100 [19].

### Statistical analysis

Data were analyzed using SPSS software (Version 22; SPSS Inc., Chicago, IL). Normally distributed data were expressed as mean ± standard deviation (SD) and data not normally distributed were presented as the median and interquartile range (IQR). The qualitative variables results were expressed as percentages. To determine the association between the 10-year risk of CVDs or BMI, with dietary intakes across different quartiles of HEI-2015, we used one-way ANOVA or Kruskal–Wallis test and linear regression. Moreover, the Mann–Whitney test was used to compare the variables between the pair groups. *P* < 0.05 was considered significant.

## Result*s*

The general characteristics of the subjects are shown in Table 1. Based on the results, 764 participants with a mean age of 48.6 ± 5.6 years were enrolled in the study. There was no significant difference between the age in men (47.1 ± 5.6) and women (46.6 ± 5.7) (*P* = 0.08), and the participants were homogeneously distributed. Height and weight values were 171.2 ± 9.2 cm and 73.0 ± 0.13 kg, respectively. Of all participants, 260 (0.34%), 366 (47.9%), and 138 (18.1%) were normal, overweight, and obese, respectively.

**Table 1:** General characteristics and QRISK3 and Framingham Risk Score of participants

**Population characteristics**	**Category**	**N (%)**	**Median(IQR)**	**Mean ± SD**	**Min**	**Max**
**Age**	Total	764 (100)	46.0 (8.0)	46.8 ± 5.6	40.0	66.0
**BMI**	Normal	260 (34)	23.4 (2.4)	22.9 ± 1.5	18.1	24.9
	Over-weight	366 (47.9)	27.2 (2.3)	27.2 ± 1.3	25.0	29.9
	Obese	138 (18.1)	31.6 (3.5)	32.8 ± 2.9	30.0	44.7
**Marital status**	Married	645 (84.4)				
**Family history of diabetes**	Yes	102 (13.4)				
**Family history of hypertension**	Yes	118 (15.4)				
**Family history of cardiovascular disease**	Yes	200 (26.2)				
**Having diabetes**	Yes	31 (4.1)				
**Current smoking**	Yes	69 (9.0)				
**Taking hypertension treatments**	Yes	54 (7.1)				
**HDL-C**	Total	764 (100)	50.52 (14.0)	51.41 ± 9.90	26	97
**TC**	Total		179.34 (43.0)	180.80 ± 33.66	83	386
**TC / HDL-C**	Total		3.53 (1.03)	3.60 ± 0.76	1.82	8.53
**FBS**	Total		92.70 (12.96)	95.16 ± 17.31	62.63	275.91
**SBP**	Total		108.50 (19.0)	110.95 ± 14.52	80.0	178.0
**DBP**	Total		74.50 (12.0)	74.97 ± 10.21	34.50	112.0
**QRISK3 10-year risk**	low	707 (96.7)	1.8 (2.1)	2.4 ± 1.9	0.4	9.9
	moderate	24 (3.3)	12.6 (2.5)	12.5 ± 1.7	10.0	15.8
**Framingham 10 years risk**	low	639 (87.7)	2.9 (3.1)	3.5 ± 2.2	0.7	9.9
	moderate	86 (11.5)	12.9 (4.6)	13.9 ± 3.0	10.0	20.0
	High	6 (0.8)	22.7 (5.9)	23.9 ± 3.5	20.2	29.7

*BMI* body mass index, *HDL-C* high-density lipoprotein cholesterol, *TC* total cholesterol, *FBS* fasting blood sugar, *SBP* systolic blood pressure, *DBP* diastolic blood pressure

The QRISK3 index demonstrated that 707 (96.7%) people were at low risk and 24 (3.3%) at moderate risk. The FRS showed that 639 (87.7%) participants were at low risk, 86 (11.5%) were at moderate risk, and 6 (0.8%) were at high risk.

Table 2 displays the median (IQR) of the HEI-2015 total score and its components across quartiles of HEI-2015. The results showed that there was a significant difference between all components of the HEI-2015 in 4 groups (*p* < 0.05). Those in higher quartiles of HEI-2015 had higher scores including intake of total fruits, whole fruits, whole vegetables, greens and beans, whole grains, dairy products, total protein foods, seafood, plant proteins (*P* < 0.001), and, unsaturated to saturated fatty acids ratio (*P* < 0.05). Intake of refined grains was heterogeneously decremental through the quartiles (*P* < 0.001). Added sugar and SFA intake increased significantly through the quartiles (*P* < 0.001); however, added sugar scores showed a decremental trend through the quartiles (*P* < 0.001).

**Table 2:** Comparison of healthy eating index components based on HEI-2015 quartiles

**Variable**	**Min–max score**	**Q1**	**Q2**	**Q3**	**Q4**	***P*** **-value**
**Adequacy**						
Total fruits^a^		0.40 (0.50)	0.70 (0.60)	0.80 (0.60)	1.00 (0.50)	< 0.001*
Whole Fruits^a^		0.40 (0.50)	0.70 (0.50)	0.80 (0.60)	0.90 (0.50)	< 0.001*
Total vegetables^a^		0.90 (0.70)	1.4 (0.90)	1.4 (0.90)	1.6 (0.8)	< 0.001*
Greens and beans^a^		0.20 (0.20)	0.30 (0.20)	0.40 (0.30)	0.40 (0.30)	< 0.001*
Whole grains^a^		0.40 (0.80)	0.80 (1.20)	1.30 (1.30)	1.70 (1.10)	< 0.001*
Dairy^a^		0.20 (0.30)	0.40 (0.30)	0.40 (0.30)	0.50 (0.30)	< 0.001*
Total protein foods^a^		1.70 (1.40)	2.70 (1.50)	2.70 (1.30)	2.70 (1.20)	< 0.001*
Seafood and plant proteins^a^		0.70 (0.80)	1.30 (0.90)	1.30 (1.10)	1.40 (1.0)	< 0.001*
Unsaturated to saturated Fatty acids ratio^b^		2.50 (1.10)	2.60 (0.90)	2.70 (0.80)	2.70 (0.60)	0.005*
**Moderation**						
Refined grains^a^		6.90 (4.40)	7.20 (4.10)	6.10 (3.80)	4.30 (4.00)	< 0.001*
Sodium^c^		0.90 (0.50)	1.00 (0.60)	1.00 (0.50)	0.90 (0.30)	< 0.001*
Added sugars, % kcal		15.8 (10.5)	22.7 (9.4)	24.6 (12.8)	25.5 (9.5)	< 0.001*
Saturated fats, % kcal		5.50 (3.50)	6.60 (3.30)	6.60 (2.30)	6.90 (1.90)	< 0.001*
**The total score of HEI-2015**	0–100	56.3 (7.1)	63.10 (2.4)	67.8 (2.4)	72.9 (3.4)	< 0.001*
**Adequacy**						
Total fruits score	0–5	2.40 (2.90)	4.50 (2.10)	5.00 (1.50)	5.00 (4.40)	< 0.001*
Whole Fruits score	0–5	4.70 (2.40)	5.00 (0.00)	5.00 (0.00)	5.00 (0.00)	< 0.001*
Total vegetable score	0–5	4.10 (1.90)	5.00 (0.40)	5.00 (0.20)	5.00 (0.00)	< 0.001*
Greens and beans score	0–5	4.80 (2.30)	5.00 (0.00)	5.00 (0.00)	5.00 (0.00)	< 0.001*
Whole grains score	0–10	2.60 (5.10)	5.20 (7.60)	8.40 (4.70)	10.00 (2.00)	< 0.001*
Dairy score	0–10	1.90 (2.20)	2.90 (2.40)	3.20 (2.40)	3.90 (2.60)	< 0.001*
Total protein foods score	0–5	3.40 (2.50)	5.00 (1.00)	5.00 (0.80)	5.00 (0.50)	< 0.001*
Seafood and plant proteins score	0–5	4.40 (2.80)	5.00 (0.00)	5.00 (0.00)	5.00 (0.00)	< 0.001*
Unsaturated to saturated Fatty acids ratio score	0–10	9.90 (3.90)	10.00 (1.50)	10.00 (1.30)	10.00 (0.50)	< 0.001*
**Moderation**						
Refined grains score	0–10	0.00 (0.00)	0.00 (0.00)	0.00 (0.30)	0.00 (0.60)	< 0.001*
Sodium^c^	0–10	10.00 (0.90)	10.00 (2.90)	10.00 (1.60)	10.00 (0.20)	< 0.001*
Added sugars score	0–10	5.20 (5.40)	2.30 (4.50)	0.70 (3.60)	0.20 (2.50)	< 0.001*
Saturated fats score	0–10	10.00 (0.00)	10.00 (0.80)	10.00 (0.20)	10.00 (0.00)	0.009*

Data expressed as median (IQR)

*HEI-2015* Healthy eating index-2015

*P* < 0.05 was considered significant

^*^Kruskal–Wallis test was used for quantitative data; Mann–Whitney tests were used to compare variables between pair groups

^a^Expressed as the total number of cup- or ounce-equivalents per 1000 kcal

^b^Calculated as (polyunsaturated fat + monounsaturated fat)/saturated fat

^c^Expressed as grams per 1000 kcal

HEI-2015 and its components were compared based on BMI categorization: normal, overweight, and obese. As the results presented in Table 3, there was a statistically significant difference between the total HEI-2015 score in the overweight and obese participants (*P* = 0.03). There was also a significant difference between the daily consumption of whole grains in the overweight and obese groups (*P* < 0.05), so the overweight subjects had a higher intake than the obese subjects.

**Table 3:** HEI-2015 and its components based on BMI, FRS, and Qrisk3

**HEI-2015 components**	**BMI**			P^**^	**FRS**			P^**^	**Qrisk3**		P^**^
	**Normal weight**	**Over-weight**	**Obese**		**Low**	**Moderate**	**High**		**Low**	**Moderate**	
The total score of HEI-2015	65.6 (9.0)	66.0 (9.3)^a^	63.6 (11.1)^a^	0.03*	65.6 (9.3)^a^	64.2 (12.3)^a^	68.4 (12.1)	0.24	65.6 (9.5)	62.3 (10.8)	0.52
**Adequacy**											
Total fruits^b^	0.7 (0.7)	0.8 (0.7)	0.7 (0.6)	0.24	0.8 (0.7)^a^	0.6 (0.6)^a^	0.9 (0.9)	0.003*	0.7 (0.7)	0.6 (0.5)	0.07
Whole Fruits^b^	0.7 (0.7)	0.8 (0.7)	0.7 (0.6)	0.28	0.8 (0.7)^a^	0.6 (0.6)^a^	0.9 (0.9)	0.003*	0.7 (0.7)	0.6 (0.5)	0.02*
Total vegetables^b^	1.3 (0.9)	1.4 (0.8)	1.3 (1.0)	0.92	1.3 (0.8)	1.3 (0.9)	1.5 (2.6)	0.27	1.4 (0.9)	1.3 (1.2)	0.92
Greens and beans^b^	0.3 (0.3)	0.3 (0.3)	0.3 (0.3)	0.45	0.3 (0.3)	0.3 (0.3)	0.4 (0.5)	0.49	0.3 (0.3)	0.4 (0.3)	0.98
Whole grains^b^	1.1 (1.4)	1.1 (1.5)^a^	0.8 (1.3)^a^	0.02*	1.0 (1.3)	1.0 (1.5)	1.0 (1.9)	0.53	1.1 (1.4)	0.9 (1.0)	0.47
Dairy^b^	0.4 (0.4)	0.4 (0.3)	0.4 (0.3)	0.53	0.4 (0.4)	0.4 (0.3)	0.3 (0.2)	0.14	0.4 (0.4)	0.3 (0.2)	0.14
Total protein foods^b^	2.5 (1.5)	2.6 (1.4)	2.5 (1.4)	0.73	2.6 (1.4)	2.4 (1.2)	2.2 (2.2)	0.31	2.6 (1.4)	2.3 (1.7)	0.37
Seafood and plant protein	1.2 (1.0)	1.2 (1.0)	1.1 (1.2)	0.57	1.2 (1.0)	1.3 (1.0)	1.3 (2.0)	0.20	1.2 (1.0)	1.1 (1.0)	0.30
Unsaturated to saturated Fatty acids ratio^c^	2.7 (0.90)	2.7 (0.8)	2.6 (0.9)	0.91	2.6 (0.8)	2.6 (0.9)	2.6 (1.6)	0.82	2.6 (0.8)	2.7 (1.3)	0.84
**Moderation**											
Refined grains^b^	6.6 (4.5)	6.1 (4.4)	5.7 (4.1)	0.05	6.2 (4.1)	6.0 (5.7)	4.2 (6.1)	0.43	6.2 (4.2)	6.7 (5.7)	0.55
Sodium^d^	0.9 (0.5)	0.9 (0.5)	0.9 (0.4)	0.80	0.9 (0.5)	0.9 (0.5)	0.9 (0.4)	0.75	0.9 (0.5)	0.9 (0.6)	0.76
Added sugars, % kcal	22.2 (11.4)	21.9 (12.2)	20.3 (11.6)	0.12	21.9 (11.5)^a^	19.5 (13.3)^a^	25.5 (17.1)	0.01*	21.8 (11.3)	21.3 (14.9)	0.35
Saturated fats, % kcal	6.8 (2.9)	6.5 (2.9)	6.3 (3.4)	0.33	6.6 (3.0)^a^	6.0 (3.4)^a^	4.9 (2.2)	0.008*	6.6 (2.9)	5.9 (4.3)	0.09

Data expressed as median (IQR)

*HEI-2015* Healthy eating index-2015, *BMI* body mass index, *FRS* Framingham risk score, *CVD* Cardiovascular disease, *HTN* Hypertension

^*^P < 0.05 was considered significant

^**^Kruskal–Wallis test was used for quantitative data and the Mann–Whitney test was used to compare variables between pair groups

^a^significance of pairwise comparisons showed with a letter

^b^Expressed as the total number of cup- or ounce-equivalents per 1000 kcal;

^c^Calculated as (polyunsaturated fat + monounsaturated fat)/saturated fat;

^d^Expressed as grams per 1000 kcal

The total fruit (*P* = 0.003), the whole fruit (*P* = 0.003), added sugar (*P* = 0.01), and SFA intake (*P* = 0.008) indicated a statistically significant inverse difference between the low and moderate Framingham risk groups.

Also, the results showed a significant difference between whole fruit consumption (*P* = 0.02) among people with low and medium risk, based on the QRISK3 risk score. There was no remarkable difference between the two groups in other variables. Also, a significant reverse association between HEI-2015 and QRISK3 and Framingham risk scores was observed (Table 4).

**Table 4:** Association between HEI-2015 and Framingham and QRISK3 Risk Scores

**Variables**	**HEI-2015**			
**HEI-2015**	**N**	**Beta** **Standardized**	**CI 95%**	P^*^
**Framingham Risk Score**				
Crude model	727	-0.105	-0.102, -0.019	**0.005**
Adjusted model	721	-0.063	-0.070, -0.002	**0.035**
**Qrisk3 Risk Score**				
Crude model	729	-0.085	-0.055, -0.004	**0.022**
Adjusted model	723	-0.057	-0.035, -0.005	**0.009**

*P* < 0.05 was considered significant

^*^Linear regression test adjusted for age, energy intake, BMI, diabetes, hypertension treatment, and last education

## Discussion

The results of our study indicated that the intake of whole fruits, whole fruits, whole vegetables, greens and beans, whole grains, dairy products, whole protein foods, seafood, plant proteins, and unsaturated to SFAs ratio had an increasing trend through HEI-2015. The intake of refined grains was heterogeneously decremental through the quartiles. Also, added sugar and SFAs increased significantly through the quartiles. Higher HEI-2015 score and whole grains intake were related to lower BMI, and higher fruit consumption was associated with lower FRS and QRISK3. However, a higher intake of added sugars and SFAs was significantly associated with lower FRS.

Various studies have recommended the protective effect of HEI-2015 against several morbidities such as depression and anxiety, metabolic syndrome, cancers, sarcopenia, etc. [20–23]. In a study by Sullivan et al., higher diet quality was related to lower SFAs and refined grains intake. Moreover, HEI was associated with more polyunsaturated fatty acids and whole grain consumption, and adherence to it favorably affected the cardiovascular risk factors [24]. Also, Manios et al. showed that lower HEI scores were associated with high saturated fat intake and low vegetable intake in preschoolers in Greece [25]. Furthermore, Saneei et al. reported that adherence to AHEI-2010 was related to higher consumption of carbohydrates, protein, fiber, n-3 fatty acids, B-complex vitamins, and lower energy intake. In addition, subjects in the fourth quartile of AHEI-2010 had higher consumption of fruits, vegetables, whole grains, dairy, and nuts with lower intakes of trans-fatty acids, refined grains, red meat, and sugar-added beverages compared to the first quartiles [22]. In line with previous studies, Jessri et al. revealed a remarkable incremental trend across quartiles of fiber, polyunsaturated fatty acids, and proteins; also, a decremental trend was observed across SFAs, alcohol, and added sugar intake [26]. However, contrary to previous studies and in line with our findings, Hooshmand et al. showed that sugar intake increased according to the HEI quartiles [27].

Diet quality, rather than quantity, can reflect comprehensive assessments of a person’s intake and affect various aspects of health [28, 29]. Saraf-Bank et al. indicated that women with the highest scores of HEI-2010 had the lowest metabolic syndrome risk and its components, including abdominal obesity, high blood pressure, high serum triacylglycerol, and low serum HDL-C [23]. It has been shown by Tande et al. that adherence to HEI is associated with lower abdominal obesity [11]. Also, Gao et al. reported that HEI could be a predictor of waist circumference and BMI [30].

Based on our results, lower HEI-2015 and whole grain consumption were related to higher BMI. Whole grains have large amounts of food compounds and nutrients, such as complex carbohydrates, fiber, vitamins (A, E, and B-complex vitamins) minerals, and phytochemicals which might have an impact on the body weight, alleviate insulin resistance, and reduction of CVDs risk [31, 32]. In this regard, Vijver and colleagues claimed that whole grain consumption had protective effects against higher BMI and getting overweight or obese, in healthy middle-aged participants [33]. Also, Harland et al. showed that people with higher consumption of whole grains were likely to have lower BMI and central adiposity [34]. Kikuchi et al. provided evidence that whole-grain wheat bread consumption, rather than refined wheat bread, led to visceral fat obesity reduction in the Japanese population [35]. Although a meta-analysis with ten cohort studies reported an inverse relationship between the whole grain consumption and risk of stroke, coronary heart disease (CHD), and CVDs [36], we did not observe a significant relationship between whole grain intake and FRS and QRISK3 scores in the current study.

According to the results of the present study, higher intake of fruits was associated with lower FRS and QRISK3 scores. Fruits have anti-inflammatory and antioxidant properties due to fiber content, various types of vitamins (folate, vitamin C, and beta carotene), minerals, and bioactive phytochemicals (polyphenols), which have been negatively associated with CVDs risk factors [37]. In addition, the flavonoid content of fruits has lipid-reducing, anti-platelet, and anti-hypertensive effects [38]. Mellendick et al. suggests that a higher intake of fruits and vegetables, and a lower intake of sweetened beverages have cardio-protective effects [39]. Another study by Buil-Cosiales et al. showed the beneficial effects of high fruit consumption on CVDs prevention in the Mediterranean population [40]. Also, Bhupathiraju and colleagues revealed that vegetable and fruit intake lowered the risk of CHD [41]. However, Bhupathiraju et al. claim that fruit variety is much more important than quantity in the prevention of inflammatory status and CHD [42].

Based on our findings, higher added sugar and SFAs intake was significantly related to lower FRS, which is contrary to previous findings. As LeungScD et al. indicated a higher intake of sugar-sweetened beverages was associated with poorer diet quality in children [43]. Moreover, Yang et al. observed that US adults had more intake of added sugar compared to healthy guidelines, which increased the mortality rate from CVDs [44]. In this regard, it can be said that although the added sugar was less consumed in the moderate-risk group compared to FRS low-risk group, in general, the sugar consumption of all three groups was much higher than the maximum recommended amount of HEI-2015. Therefore, in this condition, the relationship between the consumption of added sugar and cardiovascular risk cannot be properly assessed and the relevant results need further studies.

On the other hand, some investigations have revealed a correlation between SFAs and an increased risk of ischemic heart disease. SFAs consumption showed an increasing trend in cholesterol levels, which is an important risk factor for CVDs [45, 46]. However, a cohort study by the JACC study group in Japan showed that SFAs intake negatively correlated with stroke and intraparenchymal hemorrhage. It should be noted that SFAs intake increases HDL-C as well as LDL-C concentration, which should be considered in the net effect of SFA on CVDs [47]. Furthermore, another meta-analysis by Siri-Tarino et al. revealed no significant relationship between SFA intake and a higher risk of CVDs and CHD [48]. Even though SFAs were consumed less in the higher Framingham risk groups, according to HEI components quartiles, the average intake of all risk groups of Framingham was within the recommended range, so SFAs may not affect CVDs.

One possible limitation of the current study was its cross-sectional design, so no causal association can be obtained from the findings. Second, in case FFQ is used as a retrospective dietary evaluation tool, the risk of recall bias is probable. As to the strengths of our study, it is important to note that we used HEI-2015, which is the latest version of the questionnaire and updated based on new guidelines. Also, we used the latest version of QRISK3 and Framingham online software. The large sample size of the present study was another strength.

## Conclusion

In conclusion, the results of our study support dietary recommendations to increase fruit and whole grains intake to prevent obesity and risks of CVDs, by achieving a higher quality diet. Also, a significant reverse association between HEI-2015 and QRISK3 and Framingham risk scores was observed. However, results for added sugar and SFAs were controversial. Further studies are needed to confirm these results.

## Data Availability

The datasets used and analyzed during the current study are available from the corresponding author upon reasonable request.

## Abbreviations

CVDs: Cardiovascular diseases
WHO: World health organization
FRS: Framingham risk score
HEI: Healthy eating index
SUMS EHCS: Shiraz university of medical sciences employees health cohort study
BMI: Body mass index
HDL: High-density lipoprotein cholesterol
LDL: Low-density lipoprotein cholesterol
FFQ: Food frequency questionnaire
SFA: Saturated fatty acid
PUFA: Polyunsaturated fatty acid
MUFA: Monounsaturated fatty acid
SD: Standard deviation
IQR: Interquartile range
CHD: Coronary heart disease

## References

[1] Wang H, Naghavi M, Allen C, Barber RM, Bhutta ZA, Carter A, Casey DC, Charlson FJ, Chen AZ, Coates MM (2016). Global, regional, and national life expectancy, all-cause mortality, and cause-specific mortality for 249 causes of death, 1980–2015: a systematic analysis for the Global Burden of Disease Study 2015. Lancet.

[2] Balakumar P, Maung-U K, Jagadeesh G (2016). Prevalence and prevention of cardiovascular disease and diabetes mellitus. Pharmacol Res.

[3] Sarrafzadegan N, Mohammmadifard N (2019). Cardiovascular disease in Iran in the last 40 years: prevalence, mortality, morbidity, challenges and strategies for cardiovascular prevention. Arch Iran Med.

[4] Damen JA, Pajouheshnia R, Heus P, Moons KG, Reitsma JB, Scholten RJ, Hooft L, Debray TP (2019). Performance of the Framingham risk models and pooled cohort equations for predicting 10-year risk of cardiovascular disease: a systematic review and meta-analysis. BMC Med.

[5] Dhaliwal SS, Welborn TA (2009). Central obesity and multivariable cardiovascular risk as assessed by the Framingham prediction scores. Am J Cardiol.

[6] Elagizi A, Kachur S, Carbone S, Lavie CJ, Blair SN: A Review of Obesity, Physical Activity, and Cardiovascular Disease. Curr Obes Rep. 2020;9:1-11. 10.1007/s13679-020-00403-z 32870465

[7] Im E, Kim G-S (2017). Relationship between sleep duration and Framingham cardiovascular risk score and prevalence of cardiovascular disease in Koreans. Medicine.

[8] Ghosal S, Sinha B, Ved J, Biswas M (2020). Quantitative measure of asymptomatic cardiovascular disease risk in Type 2 diabetes: evidence from Indian outpatient setting. Indian Heart J.

[9] Aa A (2014). Diet quality concept. Nutrition.

[10] Nicklas TA, O'Neil CE, Fulgoni VL (2012). Diet quality is inversely related to cardiovascular risk factors in adults. J Nutr.

[11] Tande DL, Magel R, Strand BN (2010). Healthy Eating Index and abdominal obesity. Public Health Nutr.

[12] Gesteiro E, Bernal BR, Bastida S, Sánchez-Muniz F (2012). Maternal diets with low healthy eating index or Mediterranean diet adherence scores are associated with high cord-blood insulin levels and insulin resistance markers at birth. Eur J Clin Nutr.

[13] Xu Z, Steffen LM, Selvin E, Rebholz CM (2020). Diet quality, change in diet quality, and risk of incident cardiovascular disease and diabetes. Public Health Nutr.

[14] Esmaeilinezhad Z, Babajafari S, Sohrabi Z, Eskandari M-H, Amooee S, Barati-Boldaji R (2019). Effect of synbiotic pomegranate juice on glycemic, sex hormone profile and anthropometric indices in PCOS: a randomized, triple blind, controlled trial. Nutr Metab Cardiovasc Dis.

[15] Preston SH, Fishman E, Stokes A (2015). Effects of categorization and self-report bias on estimates of the association between obesity and mortality. Ann Epidemiol.

[16] Khanna NN, Jamthikar AD, Gupta D, Nicolaides A, Araki T, Saba L, Cuadrado-Godia E, Sharma A, Omerzu T, Suri HS (2019). Performance evaluation of 10-year ultrasound image-based stroke/cardiovascular (CV) risk calculator by comparing against ten conventional CV risk calculators: a diabetic study. Comput Biol Med.

[17] Ghayour-Mobarhan M, Moohebati M, Esmaily H, Ebrahimi M, Parizadeh SMR, Heidari-Bakavoli AR, Safarian M, Mokhber N, Nematy M, Saber H (2015). Mashhad stroke and heart atherosclerotic disorder (MASHAD) study: design, baseline characteristics and 10-year cardiovascular risk estimation. Int J Public Health.

[18] Willett WC, Sampson L, Stampfer MJ, Rosner B, Bain C, Witschi J, Hennekens CH, Speizer FE (1985). Reproducibility and validity of a semiquantitative food frequency questionnaire. Am J Epidemiol.

[19] Rahmani J, Varkaneh HK, Ryan PM, Zarezadeh M, Rashvand S, Clark C, Day AS, Hekmatdoost A (2019). Healthy Eating Index-2015 as a predictor of ulcerative colitis risk in a case–control cohort. J Dig Dis.

[20] Esmaeily Z, Tajary Z, Daei S, Rezaei M, Eyvazkhani A, Motlagh ARD, Palmowski A (2021). Association between Healthy Eating Index-2015 scores and probable sarcopenia in community-dwelling Iranian older adults: a cross-sectional study. J Nutr Sci.

[21] Kord-Varkaneh H, Salehi-Sahlabadi A, Zarezadeh M, Rahmani J, Tan SC, Hekmatdoost A, Rashidkhani B (2020). Association between Healthy Eating Index-2015 and Breast Cancer Risk: A Case-Control Study. Asian Pac J Cancer Prev.

[22] Saneei P, Hajishafiee M, Keshteli AH, Afshar H, Esmaillzadeh A, Adibi P (2016). Adherence to Alternative Healthy Eating Index in relation to depression and anxiety in Iranian adults. Br J Nutr.

[23] Saraf-Bank S, Haghighatdoost F, Esmaillzadeh A, Larijani B, Azadbakht L (2017). Adherence to Healthy Eating Index-2010 is inversely associated with metabolic syndrome and its features among Iranian adult women. Eur J Clin Nutr.

[24] Sullivan VK, Petersen KS, Fulgoni VL, Eren F, Cassens ME, Bunczek MT, Kris-Etherton PM (2021). Greater Scores for Dietary Fat and Grain Quality Components Underlie Higher Total Healthy Eating Index–2015 Scores, While Whole Fruits, Seafood, and Plant Proteins Are Most Favorably Associated with Cardiometabolic Health in US Adults. Curr Dev Nutr.

[25] Manios Y, Kourlaba G, Kondaki K, Grammatikaki E, Birbilis M, Oikonomou E, Roma-Giannikou E (2009). Diet quality of preschoolers in Greece based on the Healthy Eating Index: the GENESIS study. J Am Diet Assoc.

[26] Jessri M, Ng AP, L’Abbé MR (2017). Adapting the healthy eating index 2010 for the Canadian population: evidence from the Canadian Community Health Survey. Nutrients.

[27] Hooshmand F, Asghari G, Yuzbashian E, Mahdavi M, Mirmiran P, Azizi F (2018). Modified healthy eating index and incidence of metabolic syndrome in children and adolescents: Tehran lipid and glucose study. J Pediatr.

[28] Camhi SM, Evans EW, Hayman LL, Lichtenstein AH, Must A (2015). Healthy eating index and metabolically healthy obesity in US adolescents and adults. Prev Med.

[29] Guo X, Warden B, Paeratakul S, Bray G (2004). Healthy eating index and obesity. Eur J Clin Nutr.

[30] Gao SK, Beresford SA, Frank LL, Schreiner PJ, Burke GL, Fitzpatrick AL (2008). Modifications to the Healthy Eating Index and its ability to predict obesity: the Multi-Ethnic Study of Atherosclerosis. Am J Clin Nutr.

[31] Steffen LM, Jacobs DR, Murtaugh MA, Moran A, Steinberger J, Hong C-P, Sinaiko AR (2003). Whole grain intake is associated with lower body mass and greater insulin sensitivity among adolescents. Am J Epidemiol.

[32] Albertson AM, Reicks M, Joshi N, Gugger CK (2015). Whole grain consumption trends and associations with body weight measures in the United States: results from the cross sectional National Health and Nutrition Examination Survey 2001–2012. Nutr J.

[33] Van de Vijver L, Van den Bosch L, Van den Brandt P, Goldbohm R (2009). Whole-grain consumption, dietary fibre intake and body mass index in the Netherlands cohort study. Eur J Clin Nutr.

[34] Harland JI, Garton LE (2008). Whole-grain intake as a marker of healthy body weight and adiposity. Public Health Nutr.

[35] Kikuchi Y, Nozaki S, Makita M, Yokozuka S, Fukudome SI, Yanagisawa T, Aoe S (2018). Effects of whole grain wheat bread on visceral fat obesity in Japanese subjects: a randomized double-blind study. Plant Food Hum Nutr.

[36] Aune D, Keum N, Giovannucci E, Fadnes LT, Boffetta P, Greenwood DC, Tonstad S, Vatten LJ, Riboli E, Norat T (2016). Whole grain consumption and risk of cardiovascular disease, cancer, and all cause and cause specific mortality: systematic review and dose-response meta-analysis of prospective studies. BMJ.

[37] Holt EM, Steffen LM, Moran A, Basu S, Steinberger J, Ross JA, Hong C-P, Sinaiko AR (2009). Fruit and vegetable consumption and its relation to markers of inflammation and oxidative stress in adolescents. J Am Diet Assoc.

[38] Oh JS, Kim H, Vijayakumar A, Kwon O, Kim Y, Chang N (2017). Association of dietary flavonoid intake with prevalence of type 2 diabetes mellitus and cardiovascular disease risk factors in Korean women aged≥ 30 years. J Nutr Sci Vitaminol.

[39] Mellendick K, Shanahan L, Wideman L, Calkins S, Keane S, Lovelady C (2018). Diets rich in fruits and vegetables are associated with lower cardiovascular disease risk in adolescents. Nutrients.

[40] Buil-Cosiales P, Martinez-Gonzalez MA, Ruiz-Canela M, Díez-Espino J, García-Arellano A, Toledo E (2017). Consumption of fruit or fiber-fruit decreases the risk of cardiovascular disease in a Mediterranean young cohort. Nutrients.

[41] Bhupathiraju SN, Wedick NM, Pan A, Manson JE, Rexrode KM, Willett WC, Rimm EB, Hu FB (2013). Quantity and variety in fruit and vegetable intake and risk of coronary heart disease. Am J Clin Nutr.

[42] Bhupathiraju SN, Tucker KL (2011). Greater variety in fruit and vegetable intake is associated with lower inflammation in Puerto Rican adults. Am J Clin Nutr.

[43] Leung CW, DiMatteo SG, Gosliner WA, Ritchie LD (2018). Sugar-sweetened beverage and water intake in relation to diet quality in US children. Am J Prev Med.

[44] Yang Q, Zhang Z, Gregg EW, Flanders WD, Merritt R, Hu FB (2014). Added sugar intake and cardiovascular diseases mortality among US adults. JAMA Intern Med.

[45] Keys A, Anderson JT, Grande F (1957). Prediction of serum-cholesterol responses of man to changes in fats in the diet. Lancet.

[46] Collaboration PS (2007). Blood cholesterol and vascular mortality by age, sex, and blood pressure: a meta-analysis of individual data from 61 prospective studies with 55 000 vascular deaths. The Lancet.

[47] Yamagishi K, Iso H, Yatsuya H, Tanabe N, Date Ch, Kikuchi Sh, Yamamoto A, Inaba Y, Tamakoshi A. Dietary intake of saturated fatty acids and mortality from cardiovascular disease in Japanese: the Japan Collaborative Cohort Study for Evaluation of Cancer Risk (JACC) Study. The Am J of Clin Nutr. 2010;92:759–65. 10.3945/ajcn.2009.29146 20685950

[48] Siri-Tarino PW, Sun Q, Hu FB, Krauss RM (2010). Meta-analysis of prospective cohort studies evaluating the association of saturated fat with cardiovascular disease. Am J Clin Nutr.

